# Methods of Gold and Silver Nanoparticles Preparation

**DOI:** 10.3390/ma13010001

**Published:** 2019-12-18

**Authors:** Petr Slepička, Nikola Slepičková Kasálková, Jakub Siegel, Zdeňka Kolská, Václav Švorčík

**Affiliations:** 1 Department of Solid State Engineering, The University of Chemistry and Technology, 166 28 Prague, Czech Republic; nikola.kasalkova@vscht.cz (N.S.K.); jakub.siegel@vscht.cz (J.S.); vaclav.svorcik@vscht.cz (V.Š.); 2 Faculty of Science, J.E. Purkyně University, 400 96 Ústí nad Labem, Czech Republic

**Keywords:** nanoparticle, preparation, noble metal, surface

## Abstract

The versatile family of nanoparticles is considered to have a huge impact on the different fields of materials research, mostly nanoelectronics, catalytic chemistry and in study of cytocompatibility, targeted drug delivery and tissue engineering. Different approaches for nanoparticle preparation have been developed, not only based on “bottom up” and “top down” techniques, but also several procedures of effective nanoparticle modifications have been successfully used. This paper is focused on different techniques of nanoparticles’ preparation, with primary focus on metal nanoparticles. Dispergation methods such as laser ablation and vacuum sputtering are introduced. Condensation methods such as reduction with sodium citrate, the Brust–Schiffrin method and approaches based on ultraviolet light or biosynthesis of silver and gold are also discussed. Basic properties of colloidal solutions are described. Also a historical overview of nanoparticles are briefly introduced together with short introduction to specific properties of nanoparticles and their solutions.

## 1. Introduction

One of the most important areas of modern science is the preparation of colloidal solutions of metallic nanoparticles. Gold nanoparticles, along with their applications, are currently one of the most studied materials used in many areas, such as optoelectronics and catalysis [1]. Gold and silver nanoparticles are used in nanobiotechnology, biosensor studies, visualization of cell structures [2] and targeted drug delivery [3]. Currently, colloidal nanoparticles are studied due to their unique physicochemical properties that differ from those of “bulk” [4]. The shape or size of the nanoparticle has an important role in all nanotechnology applications [5]. Unique properties of metal nanoparticles [6] induces their applications such as fuel cells, environmental protection, the nanoslusters may be also prepared on surface of periodic pattern [7] for sensor enhancement. The basic operating principle of these applications is unique optical properties, especially localized surface plasmon resonance (LSPR) [8]. Of great importance is the study of optical properties of metal nanoparticles, stabilization and aggregation mechanisms of colloids. Solution stability is often accompanied with an external change (precipitation and discoloration), indicating increases in distribution or aggregation, which are undesirable changes. Different approaches are used to obtain particles having the desired properties, resulting in the desired particle shape or size or distribution [9]. Various approaches are used to maintain constant properties of solution of nanoparticles (electrostatic force stabilization, polymer stabilization and chemical surface treatment). The development of science in the area of nanotechnologies aims to improve these methods.

Among gold nanoparticles, also silver nanoparticles (Ag-NPs) have to be considered as very important with high-demand material for tissue engineering and antibacterial applications due to their effective antibacterial activity both in solution and in components [10]. Silver nanoparticles are beneficial in medicine and medicinal devices, biotechnology, electronics or environmental science [11]. Ag-NPs are extensively applied also in healthcare products, the food industry and clothing [12]. The Ag-NPs oxidative activity is accompanied with the release of silver ions, results in several negative effects on biological systems by inducing cytotoxicity, genotoxicity or immunological responses, and even cell death [13,14,15]. The applications of silver based materials are remarkably significant in human life and in many fields of industry and are involved in the composition of a wide variety of products and systems used in everyday life, which represent also a potential toxic effects after oral exposure [16].

## 2. Historical Overview

Colloidal metal solutions (from Greek, Kola, meaning “glue”) exhibit the best examples of nanotechnology during antiquity, the Middle Ages and the modern age. A colloidal system is a system in which one substance is in the form of particles of different size distributed in another. The continuous phase is called the dispersed medium and the dispersed material—the dispersed phase. Metallic colloids are considered as “finely divided” species of catalysts, stained glass, dyes and photographic materials, and ultimately, the “ancestors” of quantum dots [17].

**Figure 1:**
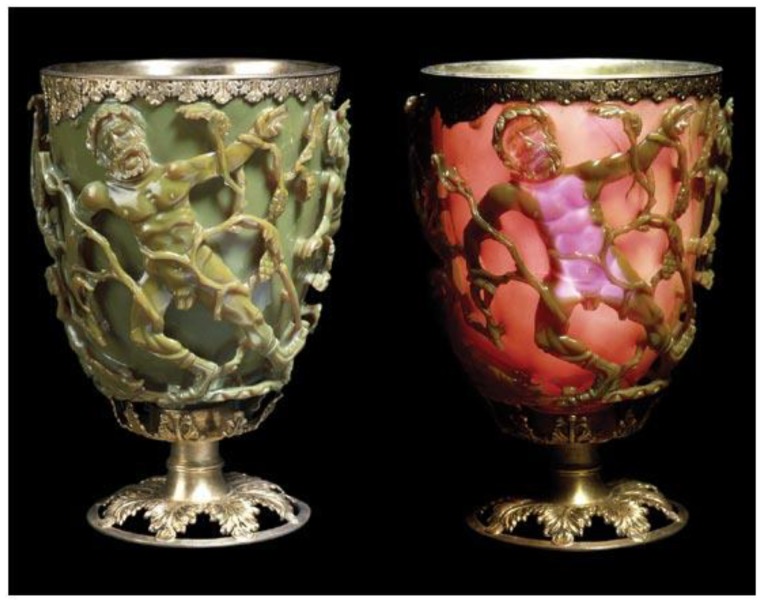
Lycurgus Cup [20].

The Lycurgus Cup is an excellent example of late Roman culture and the use of “nanotechnology” in practice. The exceptional case of Roman glassmaking dating back to the fifth century reveals one of the greatest example of “antiquity nanotechnology“. Lycurgus Cup is located in the British Museum’s collections (Figure 1). If the cup is lit from the outside, it appears green. When it shines on it from the inside, the colour turns red and only the king appears purple. Cup characterization was performed by electron microscopic methods (TEM and SEM). Scientists have found that “wonderful” dichroism is present due to the nano-particle size of Ag (66.2%), Au (31.2%) and Cu (2.6%), up to 100 nm in the size that was dispersed in glass bulk. The nanometals’ majority size was from 20 to 40 nm [17,18]. After the Romans also medieval craftsmen added metal components into the glass bulk to prepare exceptional glass windows. J. Kunckel created beautiful ruby-coloured glass in the mid-16th century by infusion of gold into glass [19].

Colloidal gold sols were described by glassmaker and alchemist Antonio Neri in 1612. In the mid-17th century, colloidal gold began to be used for the production of red ruby glass and porcelain painting. ‘Purple of Cassius’ can be described as a mixture of colloidal gold and tin (IV) oxide nanoparticles, in a glass matrix [21]. A similar example is the use of silver nanoparticles for the production of lemon-yellow glasses in the cathedrals of Europe. The formation of nanosilver in glass production took place in situ. Hans Heicher published a review focused on the application of gold for medical purposes in 1718, which describes gold solutions and their stabilization by boiled starch, an example of stabilizing gold with ligands.

**Figure 2:**
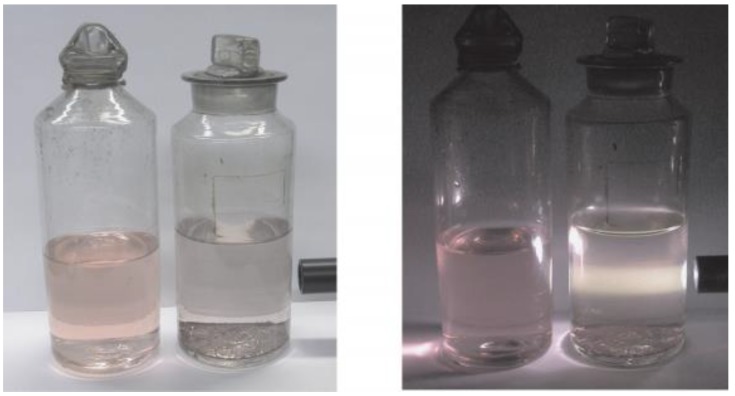
Colloidal nanoparticles [22].

Faraday synthesized gold colloid in 1857 by reducing the aqueous solution of HAuCl_4_ and described its optical properties. It is important that Faraday proved that the gold in the colloid is in a metallic state. He was also the first who reported the relationship of metallic colloids, their environment and optical properties, describing the protective effect of gelatine and other macromolecular compounds. Colloidal gold solutions prepared by him today are found at the Royal Institute in London (Figure 2):

At the turn of the 19th and 20th centuries Richard Zsigmondy described methods of the colloidal gold preparation by application of various reducing agents: hydrogen peroxide, white phosphorus and formaldehyde. Zsigmondy also suggested that the stability of the solutions is due to the charge of the colloidal particles. Together with Smoluchowski, Zsigmondy calculated what distances gold particles should be in order to aggregate them. In 1925, he was awarded the Nobel Prize for his contribution to the theory of colloidal solutions. Another Nobel laureate, Theodor Svedberg, had a number of fundamental studies devoted to the preparation and determination of the sedimentation properties of colloidal gold solutions. In his work, Svedberg used more than 25 different reducing agents and formulated a mechanism for chemical condensation of colloidal gold particles. At first, Au was considered to not be a catalyst at all, but in 1906, Bone and Wheeler showed that gold foils accelerate the reaction between hydrogen and oxygen to form water. Au gold colloidal solutions with a half-life of 65 h are used in oncology.

Currently, colloidal nanoparticles are used, for example, in photo thermal therapy. It is a promising direction in the treatment of tumours and infectious diseases. The gold nanoparticles have an absorption maximum in the visible or near infrared range when they are irradiated strongly with light of the appropriate wavelength. If nanoparticles are located inside the target cell (which can be achieved by surface modification or conjugation with the antibody [23]), the cells die. This method makes it possible to selectively destroy the tumour by photodestruction due to the thermal heating of the nanoparticles by means of laser impulses. Another strong application of nanoparticles may be found in sensors. The various types of biosensors such as enzyme-based, tissue-based, immunosensors, DNA biosensors and thermal and piezoelectric biosensors were prepared [24]. Simple and fast electron transferring processes between the analyte and sensor surface are necessary for biosensor performance. A biosensor has to simplify the formation of specific probe–target complex and triggers it into a useable reading signal. Biosensors can be classified according to the mode of physicochemical transduction or the type of bio recognition element. Electrochemical biosensors can be further classified as amperometric biosensors (that measure the current produced during oxidation or reduction of electroactive product or reactant), potentiometric biosensors (that measure the potential of the biosensor electrode with respect to a reference electrode), and conductometric biosensors. Optical biosensors rely on measurement of light absorbed or emitted as a consequence of a biochemical reaction, and are based on various optical techniques such as absorption, fluorescence, luminescence or surface plasmon resonance. Thermal biosensors are based on measurement of the thermal changes occurring on biochemical recognition. Piezoelectric biosensors involve the measurement of mass change occurring as a result of biomolecular interaction [25]. Receptors of molecules that have affinity for the proteins and DNA analysed are “constructed” on the surface of nanoparticles. Gold is used as a backing layer because it is an inert material. If the solution in which the modified nanoparticles are located contains target molecules, interactions occur, resulting in an amplification of the electrical bilayer. Surface plasmon resonance (SPR) methods were used to determine this moderate amplification [26].

## 3. Basic Properties of Colloidal Solutions

Nanoparticles can be fundamentally categorized into major two groups, organic and inorganic. Organic nanoparticles may include carbon nanoparticles [27,28] while some of the inorganic nanoparticles may include magnetic nanoparticles, noble metal nanoparticles (like gold and silver) and semiconductor nanoparticles. The approaches for synthesis of these two groups differ greatly as organic materials require relatively milder conditions of temperature, pressure and moderate pH, inorganic materials are able to withstand more extremes of these parameters. If we are speaking about the category of organic materials, polymeric and non-polymeric materials require different treatment methods. The classification of the various methods that have evolved over the years for the preparation of nanoparticles of these different categories of materials will be further presented. The application of a particular method usually depends on the properties of the material used, type of nanoparticles and their properties, also with consideration of the final application. As the method of preparation is chosen, the effect of the different parameters that may affect the process is studied. On the basis of these studies, selection of those parameters will give the best possible characteristics for the desired nanoparticles. Commonly studied nanoparticle’s parameters include size, zeta potential (surface charge) or capture/release characteristics. The technique used, type of polymer selected and stabilizer used influence the properties of the prepared nanoparticles.

As usually introduced for a nano-object, two basic approaches are used for synthesis of nanoparticles, bottom-up and top-down methods. The bottom-up technique involves building of nanostructures atom by atom (same with molecule) and involves precipitation or condensation of the product material dissolved in solvents, also with subsequent separation of unwanted solvents. The top-down method is based on size reduction of larger particles using equipment suitable for material removal form the “bulk” to construct nanosize range structures. For the equipment, devices such as sputter coater for physical vapour deposition (PVD) technique, high vacuum chamber for evaporation or laser devices for pulse laser deposition are necessary. The bottom-up approach involves a gradual build-up of the nanoparticle from individual atoms or molecules [29]. The process of a nanoparticle’s growth can take place either in the liquid, gas or vapour phase. The bottom-up techniques are capable of preparation of smaller nanoparticles with better control over the size, but are based on a specific conditions (e.g., temperature) compared to top-down techniques. For liquid phase methods, the size of the nanoparticles depends mainly on the degree of control over the precipitation or phase separation of one of the components from the solution. This may be a result of a chemical reaction or change in solubility on the basis of change in parameters such as pH or temperature with also possible effects of the solvent system. The different methods of preparation of nanoparticles using this approach emerge from the mechanism responsible for phase separation of the desired component. The factors leading phase separation and the individual process parameters finally affect the size of the prepared nanoparticles. Therefore the understanding of the theoretical aspects involved in phase separation from homogenous and heterogeneous systems is an important factor for the selection of an optimal process and modification of the physical properties of the synthesized nanoparticles. The theory of colloid stability, although a very important topic, is outside the scope of our paper.

### 3.1. Optical Properties

**Figure 3:**
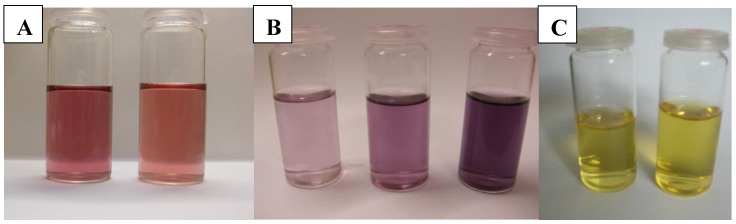
Colloidal gold (**A, B**) and silver (**C**) prepared by sputtering of metal into liquid (polyethylene glycol, PEG 600, 2 mL) and with subsequently added distilled H_2_O (18 mL).

Optical properties can be investigated by studying the interaction of light with matter. Electron behaviour of bulk materials differs from electron behaviour in nanoparticles [30]. Light scattering and absorption also have a number of peculiarities. For example, colloidal nanoparticles may have a different colour due to specific interactions with incident light. One characteristic of colloidal solutions may be the colour that changes, for example, gold nanoparticles ranging from ruby to violet to blue (Figure 3), and silver nanoparticles are characterized by a yellow colour.

**Figure 4:**
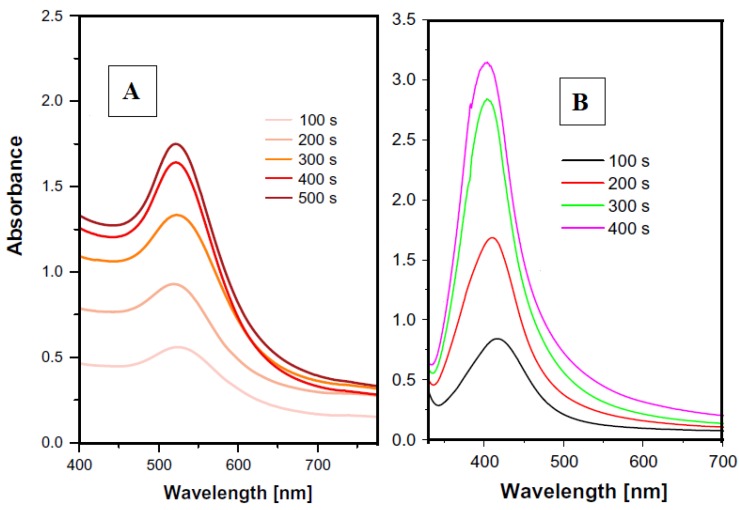
Typical UV-VIS spectra of gold (**A**) and silver (**B**) nanoparticle solution (solution in PEG/H_2_O), numbers refer to the sputtering time into PEG [33].

The dimension of nanoparticles is usually comparable to the mean free electron pathway. The mean free electron path for Au and Ag is 40–50 nm. LSPR arises not only on metal nanoparticles, but also on sharp “metal needles” and roughened metal surfaces. It can be applied in optical microscopy, fluorescence or Raman microscopy [31]. Plasmon resonance can be described as coherent excitation of the free electrons of the conductive band, which leads to their oscillation in the same phase. It can be detected if the size of the metal nanocrystals is smaller than the wavelength of the incident radiation. This phenomenon usually occurs with noble metal nanoparticles (gold and silver), as well as alkali metals and copper. For Au, Ag and Cu, the absorbance is in the visible region [32]. The model absorption spectra of gold and silver nanoparticles are introduced in Figure 4. The detailed description of interaction of nanoparticles sputtered in PEG can be found in [33]. It can be summarized, that with increasing sputtering time of metal into the liquid the concentration increases, thus leading to the increasing intensity of peak in the UV-VIS spectra. The size of the nanoparticles remains almost unchanged, therefore the peak positions are not shifted to higher wavelengths

**Figure 5:**
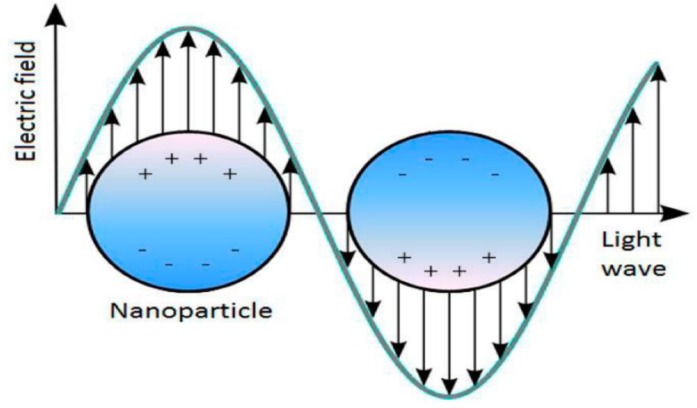
Plasmon resonance [34].

Absorption intensity depends on the concentration of metal nanoparticles in colloidal solution. For spherical nanoparticles, one peak appears (Figure 4). Schematically, plasmon resonance of spherical nanoparticles is shown in Figure 5.

Figure 5 represents the interaction of the electric field of incoming light with free electrons. The electric field polarizes free electrons. As a result, charges occur on opposite sides of the nanoparticle, resulting in a restoring force. There is a dipole oscillation of free electrons of similar frequency. The plasmon resonance energy is changed by the free electron density, particle size, shape, orientation in space and the environmental dielectric constant [35]. The larger the particles, the greater the proportion of higher-order modes because light cannot polarize the particle homogeneously. These higher order modes have maximums at lower energies. As a result, the plasmon zone is shifted towards the red region with increasing particle size and the width of the plasmon zone increases with particle size. An increase in both the wavelength in the absorption maximum and the bandwidth with increasing particle size is confirmed by experiment. This direct dependence on particle size refers to the actual magnitude of the effects [36]. When electrons collide elastically with the surface, coherence is disrupted. The smaller the size of the nanoparticle, the faster the electrons will reach the surface and the faster they will scatter. As a result, width of the plasmon resonance peak increases with increasing particle size. The advantage of spectrophotometric measurements lies in their rapidity, simplicity, relatively low running costs and versatility, and they can be used to determine the size of nanoparticles [37]. The Mie scattering theory gives an exact solution for the particle size with spherical, isotropic and homogeneous particles. Such a theory is useful in applications when the wavelength is comparable to the particle diameter and scattering is dominated by a single-scattering process in the liquid [38]. Plasmon resonance of metal NPs may be used in surface enhanced Raman scattering (SERS), mostly for spectroscopic characterization of biological and catalytic materials [39].

### 3.2. Stability of Solutions

Stabilizers are used to preserve the properties of solutions of colloids. They are substances that prevent the spontaneous coagulation of the particles. Non-stabilized particles may aggregate, preventing their further use. Silver nanoparticles have a high affinity for oxygen [40]. In the case of non-stabilized silver nanoparticles, two processes simultaneously take place—aggregation and oxidation [41]. In this case, partially oxidized nanoparticles are formed which have chemisorbed ions on the surface. Stabilization can be accomplished in a variety of ways, e.g., electrostatic repulsion, steric hindrance, nanoparticle encapsulation (such as forming a micellar layer on the surface, surrounded by thiol groups, ligands, steric shielding with large functional groups, polymeric or dendrimer) [42]. The ions form boundary layers between the dispersion medium and the dispersed phase. Macromolecular substances adsorb on the surface of nanoparticles, creating a mechanical barrier that is against aggregation.

Stabilization efficiency depends on the physico-chemical parameters of the polymer [43], its concentration, its adsorption to the surface of the nanoparticles, its solubility in the liquid medium. The applied polymer layer can decrease the effect of interfacial tension with a continuous phase interaction with the dispersion medium. Binding to macromolecules of polymeric nanoparticles can lead to selective growth of one of its sides. Different crystal facets of metal or metal oxide NP can be stabilized resulting in shape control of metal nanoparticles [44,45,46,47]. Full coverage of polymer nanoparticles will prevent component diffusion. However, an excess of stabilizer may drive the molecules to form a second layer, which also reduces the aggregate stability of the system [48]. Insufficient stabilizer results in multiple nanoparticles being adsorbed onto the surface of a single macromolecule, resulting in flocculation. The Ag and Au antibacterial activity depends on the particle size, where the formation of Ag aggregates decreases its antibacterial properties [40].

### 3.3. Antibacterial Effects of Silver and Gold

The antibacterial properties of silver have been known since antiquity [49]. Colloidal solutions of metal nanoparticles, such as Au, Ag or Cu have distinct bactericidal properties. Antimicrobial preparations based on silver nanoparticles are well established in medicine. The bactericidal properties of Ag are associated with the nanoparticles’ slow oxidation and release of Ag^+^ ions [41]. At low concentrations, nanosilver is effective against most viruses and bacteria, and microorganisms are non-likely to gain silver resistance in the mutation process because its ions attack large amounts of protein in cells. This feature is important because there are a large number of bacteria that have antibiotic resistance [50]. Nanoparticles have a larger surface area compared to bulk material, provide greater membrane permeability and can affect intracellular processes, e.g., due to presence in ion form [51]. The antibacterial effect of Ag nanoparticles depends on their size, shape and inhibition of DNA replication, manifested differently for different particle shapes [52]. Importantly, smaller nanoparticles exhibit better antibacterial properties. Smaller nanoparticles have a larger specific surface area that explains the increased biological activity of particles <10 nm in size. Based on method [53], in work [54], colloidal solutions of gold nanoparticles were prepared and their effect on various types of pathogenic Gram-positive and Gram-negative bacteria (*P. aeruginosa*, *K. oxytoca*, *E. faecalis*, *K. pneumoniae*, etc.) was studied. The results were compared with the effect of the antibiotic tetracycline. In almost all cases, gold nanoparticles showed growth in the inhibition zone: highest against *E. faecalis* (11 mm), lowest against *K. pneumoniae* (6 mm). For *E. Coli*, Au nanoparticles are more effective than antibiotics. Antibacterial effects have been observed also for silver on polyethyleneterephthalate [55], polystyrene [56] or other surface modified substrates [57].

## 4. Methods of Nanoparticles Preparation

There are physical, chemical, photochemical and biological methods of nanoparticle preparation. Further, methods of preparation of colloidal nanoparticle solutions can be divided into dispersing and condensing. Dispersion methods are based on destruction of the crystal lattice of the material (laser ablation, cathode sputtering and electric arc dispersion), it belongs to the type “top-down”. Condensation methods are based on the chemical reaction (reduction in solution, followed by the nanoparticle precipitation, formation and stabilization). Each method has its advantages and disadvantages. The modified Turkevich method results in monodisperse nanoparticles, the size of which varies depends on the reducing agent concentration and also the size of the ligand which it stabilizes [58]. By other methods, stabilization of the nanoparticles is accomplished by forming an organic monolayer on the growth surface, controlling the size and shape by the concentration of the reducing agent and the stabilizer. Also, the reducing agent may be a stabilizer [59]. Sodium citrate, alcohols, Na_2_S, borohydrides [B_2_H_6_] and sodium borohydride [NaBH_4_], including hydrogen gas [17] and sugars (fructose, glucose and sucrose) can be used for the reduction process [60]. White phosphorus and hydrazine can be used, however, these compounds are very toxic and solutions obtained by these methods cannot be used in biological applications. Recent advances in the approaches of preparation of selected noble metal nanoparticles are introduced in Table 1.

### 4.1. Dispergation Methods

#### 4.1.1. Laser Ablation

**Figure 6:**
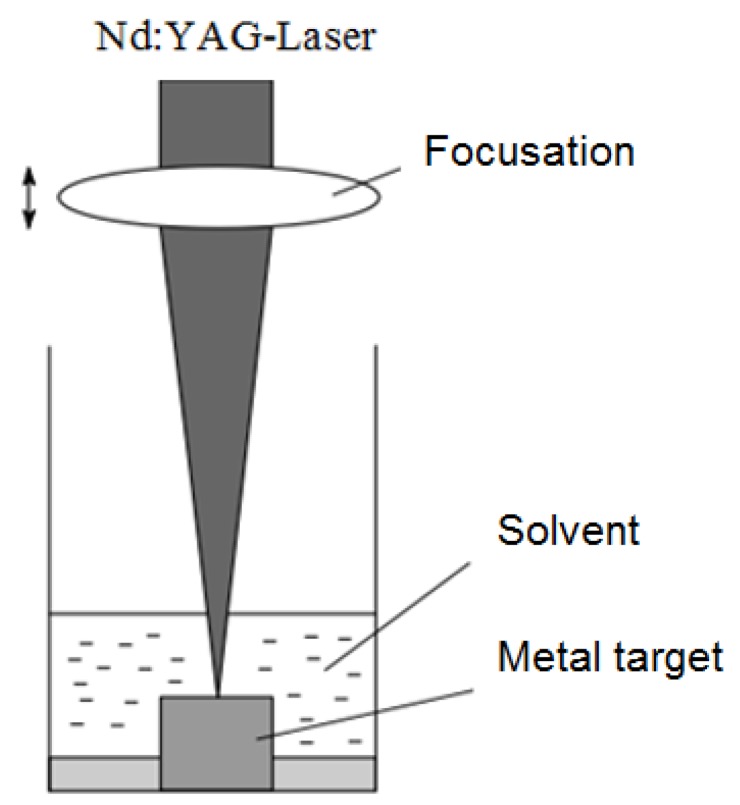
Laser ablation—preparation of Ag nanoparticles [80].

This method differs from traditional methods of nanoparticle preparation. The method is based on the irradiation of a silver sheet immersed in a surfactant or water solution by a pulsed laser. Mafuné et al. presented spherical Ag nanoparticles with 5–30 nm distribution [80]. Ag nanoparticles were prepared by ablation of an Ag plate by a laser in an aqueous solution of dodecyl sulphate, C_12_H_25_SO_4_Na, are referred to as SDS. Water is used as a medium for the nanoparticles’ formation. In the above work, a Quanta-Ray GCR-170 Nd: YAG laser was used, with a wavelength of 532 nm and 10 Hz frequency. The scheme is shown in Figure 6. The surfactant interacts with the particles formed. The nanoparticles’ size accomplished by the laser ablation method and their distribution depend on the laser intensity. Increasing intensity leads to increased size and distribution. Laser ablation modes influence the different nanoparticle shapes which may be formed. The formation of spherical nanoparticles indicates a vapour–liquid–crystal condensation mechanism.

#### 4.1.2. Vacuum Sputtering

**Figure 7:**
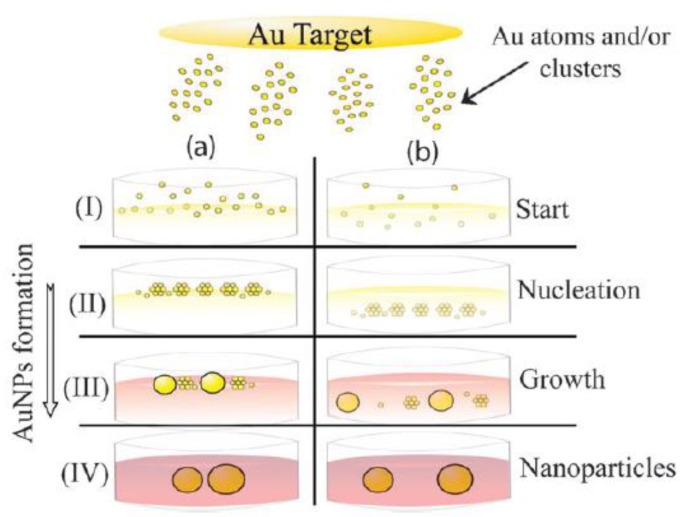
Vacuum sputtering into the liquid—preparation of nanoparticles, (**a**) nucleation starts at the oil/vacuum interface and (**b**) at the bulk liquid phase [84].

This approach is based on application of a potential difference between the two electrodes in the vacuum chamber with an electric field. An inert gas enters the chamber and is ionized [63]. The bombardment of a metal target (cathode) with argon plasma takes place. Consequently, atomic clusters are punched from the target area and deposited on the surface or into a liquid solution. The principle of solution preparation is shown schematically in Figure 7.

One of the favourite properties of this method are the simplicity and purity of the process [63]. No further chemical reactions and or application of additive stabilizers participates on the process [81]. Ionic liquids may have stabilization effect on the solution in combination with low vapour pressure [82]. The use of ionic liquids allows for preparation of the system of metal nanoparticles, reducing consecutive stabilizers [83]. Low vapour pressure is a prerequisite for the preparation of solutions by cathode sputtering. Preparation of nanoparticles by the sputtering method onto ionic liquids (ILS) is a relatively new field of research that began in 2006 with the pioneering study of Kuwabata group. Gold nanoparticles (AuNPs) with a diameter of 5.5 nm and a deviation of 0.9 nm were obtained by sputtering, directly on the surface of 1-ethyl-3-methylimidazolium tetrafluoroborate (EMI.BF_4_). In their work [84], castor oil was used as an ionic liquid. Vanecht et al. [85] realized deposition on imidazolium, but aggregates of >200 nm were formed in 24 h due to coalescence. The effect of water and ionic liquid anions on system stability has also been studied. The works in which PEG was used as a medium [86], as well as the effect of temperature, distance, time, deposition on nanoparticle formation were interesting [87]. Studies have shown that the solutions parameters obtained in this way depend on the capture medium and conditions of deposition.

The often applied media for sputtering technique are ion liquids (ILs) and significantly less-toxic vegetable oils. One of the pioneer metal depositions onto a liquid substrate was realized in 1996 by magnetron sputtering of Ag into pure silicon oil [88]. The two stages of process were described as: (a) percolation threshold and (b) first metal clusters nucleation on the liquid surface. Consequent spreading of individual clusters spread take place on the surface of liquid media, interconnections are formed which lead to continuous Ag coverage “floating” on the surface of the oil medium. As discussed earlier, the whole process strongly depended on deposition conditions, particularly sputtering power—for values less than 30 W, no Ag layers appeared to form. The preparation of ultra-thin Ag layers on the liquid surface was presented in [89].

An option to use liquid substrates for metals sputtering are vegetable oils, due to their common availability, cheapness, biocompatibility and ability to stabilize metal NPs [84] (e.g., on the basis of chemical reduction [90]). No other reducing agents which may be toxic are required, thus supporting in vivo applications. Sputtering of gold onto castor oil surface led to formation of biocompatible gold NPs. The higher voltage the larger particles have been prepared, on the other hand the increase in deposition time had almost no impact on the size of nanoparticles, which was confirmed by transmission electron microscopy (TEM) and small angle X-ray scattering (SAXS) [89]. Wender et al. [91] introduced an interesting paper where the properties of specific oil used and the deposition conditions are correlated, particularly the formation of surface thin layers on oil being in the focus of the correlation. It was shown silver nanoparticle formation depended significantly on the applied voltage and specific surface coordination ability of the media used in the experiment (canola, castor or kapron oil). If the voltage was low in combination with weak coordination ability the formation of continuous layer on the surface of oil was observed. On contrary, increase of voltage in combination with strong coordination ability led to formation of nanoparticles. Higher voltage represents higher diffusivity of impacted particles (mostly atoms of metal) liquid surface which enables: (a) penetration of particles into the liquid volume and (b) anchoring of atoms/clusters to potentially present functional groups. Silver NPs were easily formed in castor oil (solely hydroxyl groups present) in the wide interval of applied voltages. However, if canola oil is used (unsaturated aliphatic chains are mainly present) and kapron oil, thin Ag layers were deposited surface of oil for lower voltages, while AgNPs were formed at higher voltages.

More aggressive media make it possible for nanoparticle preparation (from the toxicity point of view) considered for potential application in biomedicine are already mentioned ILs [92]. Particularly, the most wide spread ILs are derivatives of organic molecules, such as pyrrolidine, imidazole or pyridine [93]. A relatively high speed of the nucleation process may be achieved during the sputtering process, thus the formation of small NPs is achieved, also without undesirable perturbations (Ostwald ripening) [94]. Moreover, these liquids can very easily change their molecular arrangement so that adaptation to nucleation centres of emerging NPs can take place supporting their stabilization [95]. Several authors consider the surface tension and liquid viscosity as critical in the formation of metal nanoparticles in liquid media. However, some authors [93] also published that in particular for the ILs, other parameters (e.g., composition and the coordinating ability of the applied liquid) play also a significant role. There is also a theory of nanoparticles growth in the volume of liquid, where an increase of nanoparticles size with increasing volume (size) of ILs anionic part may be expected [96,97,98,99]. Also, it appears that the difference in size of the prepared particles is hard to correlate with macroscopic properties of ILs, such as viscosity or surface tension [95]. The nanoparticles prepared as solutions may be used in different applications on the basis of surface grafting on polymer substrates [100,101,102,103], mostly used in tissue engineering [104,105,106,107] or for construction of antibacterial substrates [108].

### 4.2. Condensation Methods

There are currently many ways to synthesize gold nanoparticles in aqueous (solution or wet) methods, which vary according to experimental conditions and allow nanoparticles of the desired shape and distribution to be obtained [109]. In general, a high reduction force ensures a high reaction rate and thus the formation of smaller nanoparticles. Weak reducing agents cause a low reaction rate and therefore the formation of large nanoparticles. However, slow reactions may lead to an increase or decrease in nanoparticle distribution. If new nuclei are formed during the reaction, the solution will have a large distribution. By changing the conditions of the same synthesis it is possible to achieve different distribution of nanoparticles. In addition, reducing agents have a significant effect on the shape of nanoparticles [110]. As a very important technique, electrochemical methods have to also be considered, e.g., extremely small Pd nanoparticles have been successfully prepared by this technique in [111]. The electrochemical preparation can be also applied for other noble metals, such as silver [112].

#### 4.2.1. Reduction in Solution

**Figure 8:**
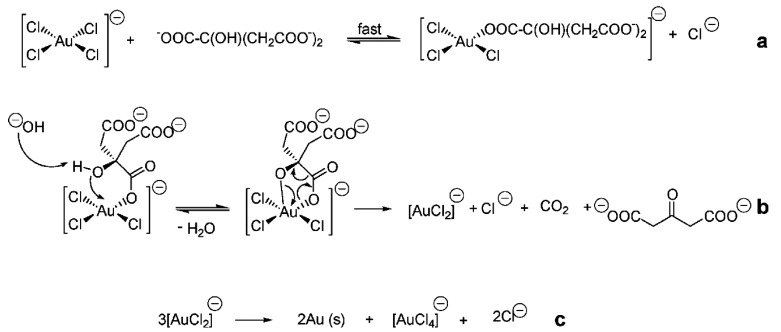
Mechanism of gold reduction [114]. Ligand exchange reaction (**a**), decarboxylation and reduction of Au(III) species (**b**) and disproportionation of aurous species and the subsequent formation of Au (O) atoms (**c**).

One of the most important methods is the use of reducing agents such as sodium citrate. This method was first proposed in 1951 by Turkevich for the preparation of monodisperse colloidal gold solutions [113]. This method makes it possible to obtain spherical nanoparticles with a size of 5–40 nm. Later this method was used for the preparation of silver nanoparticles, however, the silver nanoparticles obtained by this method had a larger distribution of 60–200 nm. Classical conditions for synthesis are: dissolution of chloroauric acid in distilled water to prepare 20 mL of a very dilute solution having a concentration of 2.5 × 10^−4^ M. This solution is brought to the boil, then 1 mL of 0.5% sodium citrate solution is added to the solution. Maintain the temperature of the reaction mixture at 100 °C until the colour changes. The solution prepared in this way has a dispersion of ~20 nm. The mechanism of formation of colloidal nanoparticles by reduction in aquatic-organic environment is represented in Figure 8.

**Figure 9:**
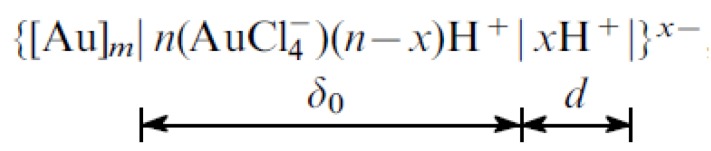
Chemical scheme of colloidal nanoparticle.

As a result, gold nanoparticles are formed on which surfaces are adsorbed ions. [AuCl_4_]^−^ forms the inner layer of the electric bilayer and determines the negative charge of the colloidal particles [115]. Chemical scheme of nanoparticle colloidal system is introduced in Figure 9.

[Au]_m_—metal core, m—number of atoms it consists of, n—number of adsorbed ions AuCl_4_¯ n < m, δ_0_—thickness of adsorption layer and d—diffuse layer thickness [116].

The disadvantage of this method is that the citrate anion acts as a reducing agent and stabilizer, which complicates the selection of the optimum citrate ratio and has a major influence on the nucleation and particle growth processes [117]. Also, oxidation products can be absorbed on the surface of the particles. Also other chemicals as reducers have been successfully used for nanoparticle’s preparation, such as hydrogen peroxide [118], sodium borohydride (NaBH_4_) [119] or ascorbic acid [120].

#### 4.2.2. Brust–Schiffrin Method

It represents the synthesis of hydrophobic gold clusters, 1–3 nm in size, stabilized by an alkanethiol monolayer in a two-phase aquatic-organic environment [121]. The aim of the synthesis is the spatial separation of nanoparticles so that they are in two immiscible phases. The organic layer prevents diffusion, diffusion is a rate limiting step in the process. The reaction rate is limited to the interface (aqueous-organic medium). Separation and hydrophobization is due to the formation of an alkanethiol monolayer which is located in a non-polar environment. Brust and Schiffrin used toluene as the non-polar medium, and tetra-n-alkylammonium was used as the interfacial carrier [122]. The synthesis of silver nanoparticles by the Brust–Schiffrin method is also possible [122].

This technique is based on the reduction of Au^3+^ complex with NaBH_4_ stabilized with thiols. The technique allows to prepare highly stable nanoparticles with narrow distribution and with possibility of their size control. Ag nanoparticles were prepared on the basis of reduction reactions [113,123]. Due to specific requirements on newly synthesized nanoparticles, numerous techniques based on both wet and dry processes were studied recently. Most of the wet-based techniques for the nanoparticle preparation exploit possibility of reaction area inside a cavity where metal ions are reduced to metals of zero valence forming nanoparticles. The sizes of the reaction cavity influences the dimension and size distribution of the prepared nanoparticles. This method has also been successfully applied for other noble metal nanoparticles, such as copper [124], where modification of this method was applied to prepare lauric acid-capped copper nanoparticles. Silver nanoparticles were prepared by modification of the Brust–Schiffrin method and subsequently these nanoparticle have been applied for construction of an electrochemical enzymeless probe [125].

#### 4.2.3. Synthesis in Reverse Micelles

**Figure 10:**
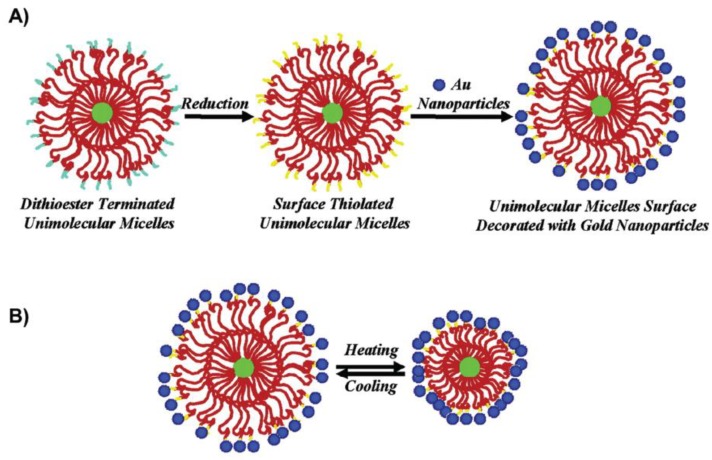
(**A**) Schematic illustration of the two-step preparation of the hybrid unimolecular micelle surface decorated with Au nanoparticles and (**B**) the thermo-tunable spatial distance between Au nanoparticles attached at the unimolecular micelle surface [126].

This method allows synthesis of nanoparticles in a confined space together with the ability to control their growth. The micro emulsion used in this method is a thermodynamically stable dispersion of two immiscible liquids that have been obtained with an emulsifier and a surfactant. Surfactants are organic molecules having different polarities. One part of the molecule is a hydrocarbon chain, which is non-polar and hydrophobic, the other part—polar. Compared to reduction in molecular solution, nanoparticles are formed in the polar core of the micelle (Figure 10). They use the water-in-oil method for metallic nanoparticles. In this case, the water is dispersed in an organic solvent. Micelles may not be used only for nanoparticle’s synthesis, but they can be also constructed as a unipolar surface decorated with Au nanoparticles [126]. Other interesting information about this approach can be found in papers [127,128].

In this paper [129], a water-in-oil colloidal solution of gold nanoparticles of 8–10 nm was obtained. The source of the Au^3+^ ion was the KAuCl_4_ salt with the reducing agent—potassium sulphite (K_2_SO_3_). Bis-(2-ethylhexyl) sulfoccinate (AOT) was used as the surfactant isooctane solvent. A 0.5 M AOT/isooctane solution was prepared. The degree of hydration ω ([H_2_O]/[AOT]) was 10. The method is based on the reduction reaction of gold ions according to the equation:

(1) $$2\;\mathrm{Au}3^{+}\;+\;3\;{\mathrm{SO}}_{3}^{2-}\;+\;3\;H_{2}O\;\to \;2\;\mathrm{Au}O\;+\;3\;{\mathrm{SO}}_{4}^{2-}\;+\;6\;H^{+}.$$

PEGylated Au nanoparticles that differ in their polyethyleneglycole molecular weight, using PEG 550 and PEG 2000 were prepared in [130]. The preparation of the gold nanoparticles was conducted by modified Brust method with a result in diameter of 4–15 nm. The folic acid has been introduced by the covalent link. This reaction is characterized by a change in colour from transparent to dark red or violet. After calibration, using standard-sized gold nanoparticles, the position of the plasmon resonance peaks was correlated with the nanoparticle size.

**Figure 11:**
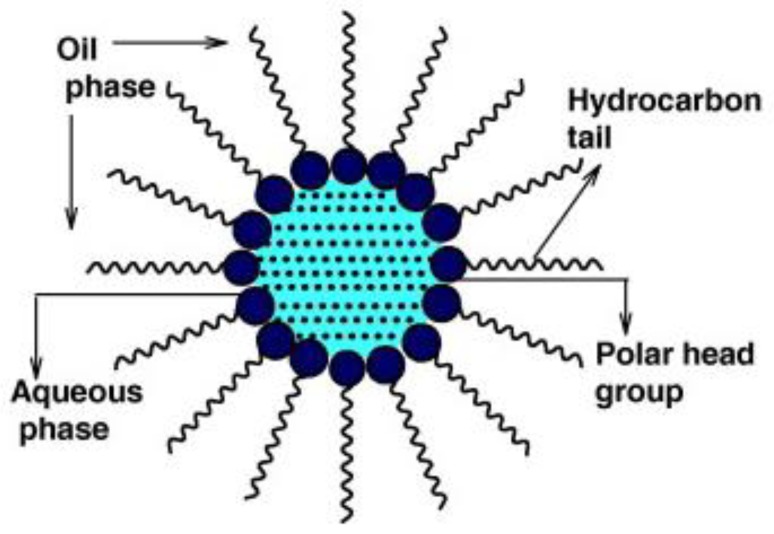
A typical structure of reverse micelle [133].

In this paper [131], a method of producing silver nanoparticles in micelles by reducing AgNO_3_ with NaBH_4_ reducing agent is presented. 2-Hydroxy-1,3-bis (octadecyldimethylammonium) propane dibromide (18-3(OH)-18) was used as a surfactant. Advantages of the surfactant are low critical micellar concentration and high solubilisation [132]. The reduction reaction is carried out in (18-3 (OH)-18)/n-heptane-1-butanol/H_2_O over 2 h with stirring. The reactions resulted in micelles containing silver nanoparticles. The nanoparticles obtained in this manner demonstrated an absorption peak of 409 nm and had a size of 12 nm in diameter. Water swollen micelles dispersed in oil as for water–in-oil (W/O) micro emulsion may be also used (Figure 11) [133]. Synthesis is reverse micelles can be easily used for the preparation of core-shell nanoparticles [134].

#### 4.2.4. Method Based on Ultraviolet Light

The synthesis of silver nanoparticles can be performed by ultraviolet irradiation of aqueous solutions containing AgClO_4_ or NaAuCl_4_, acetone, 2-propanol and various polymeric stabilizers. As acetone excites in ultraviolet radiation, ketyl radicals are generated [135]:

CH_3_COCH_3_ + (CH_3_)_2_CHOH = 2 (CH_3_)_2_(OH)C. (2)

Different approaches have been implemented for silver nanoparticles preparation by using ultraviolet light. Ultrasmall AgNPs with different optical properties were synthesized by a UV-irradiation method using chloramine T as an organic modifier [136]. Ultraviolet (UV) irradiation with UVA (320–400 nm) or UVB (280–320 nm) beam was used for changes of mobility and dissolution of citrate-coated silver nanoparticles [137]. Colloidal silver nanoparticles we also prepared by UV-light induced citrate reduction technique for the quantitative detection of uric acid [138]. Ultraviolet light has been also used for green synthesis of pullulan mediated silver nanoparticles [139].

#### 4.2.5. Biosynthesis of Silver and Gold Nanoparticles

The development of science has led to the need to develop methods for the preparation of environmentally friendly nanoparticles for the synthesis of non-toxic biocompatible nanoparticles. In 1999, a report [140] on the implementation of intracellular synthesis using the bacterial strain *Pseudomonas stutzeri* AG259 addressed this topic. It has been found that polyhedral silver crystals with an average size of 100–200 nm are produced during cell growth in the presence of Ag^+^ ions, in the periplasmic space of the cells. The mechanism of formation has not been elucidated, but it has been assumed the role of proteins that have an affinity for silver, some parts that can act as nucleation centres.

**Figure 12:**
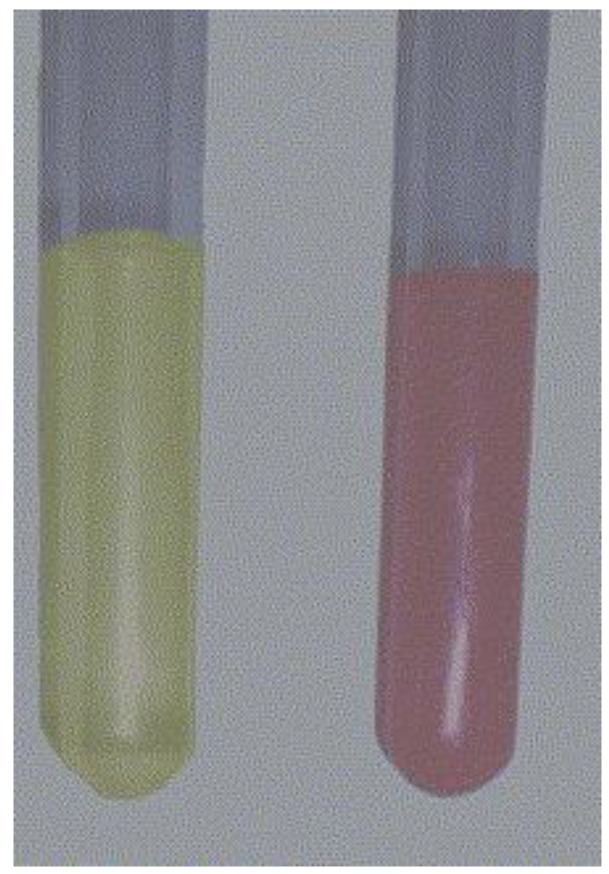
Biosynthesis of gold nanoparticles [53].

As a result of intracellular synthesis, silver nanoparticles were obtained during the growth of *Verticillium* AAT-TS-4 with an average size of 25 ± 12 nm [141]. Despite the fact that the silver NPs were immobilized in the cell walls, a hypothesis was raised about the extracellular mechanism of silver nanoparticle synthesis. Metal clusters were formed by reducing Ag^+^ ions with proteins belonging to the cell wall. Recently, *Fusarium oxysporum fungi* have been shown to be capable of intracellular reduction of aqueous silver nitrate solutions to produce metal nanoparticles of 20–50 nm in diameter. Nanoparticles demonstrated absorbance at 415–420 nm, and their aqueous dispersions demonstrated stability over several weeks. The intracellular reduction mechanism has also been confirmed [142]. *Klebsormidium flaccidum* cells demonstrated the possibility of preparation of gold nanoparticles by enzymatic method. This method is also one of the so-called green syntheses. The article by Singaravelu et al. [53] presents a method of extracellular synthesis of highly stable spherical gold nanoparticles 5–15 nm obtained by biotransformations using various types of algae (Figure 12). In a 500 mL Erlenmeyer was mixed a 10^−3^ M HAuCl_4_ solution of 100 mL with 1 g of algae powder (*S. wightii*). After further stirring for 12 h, the gold ions were reduced to AuO.

## 5. Conclusions

The historical overview of the preparation of metal nanoparticles was described in this paper. The basic properties of a nanoparticle’s colloidal solution was briefly introduced, mainly the specific properties arising from the dimension of nanoparticles and their interaction with an electromagnetic field. The most common approaches for nanoparticle colloidal solution have been introduced, the specific attention was devoted to various approaches for preparation of noble metal nanoparticles by various physico-chemical methods. Synthesis is reverse micelles can be easily used for the preparation of core-shell nanoparticles. The reduction of sodium citrate delivers stable uniform nanoparticles with a high yield. Methods based on physical vapour deposition, such as sputtering or laser ablation requires relatively expensive apparatus, but the variability of the preparation is large, and a wider spectrum of nanoparticles may be prepared, especially in the case of PVD techniques, where less chemicals may also be used. The small nanoparticles were prepared by the Brust–Schiffrin method or its modification. The green methods based on biosynthesis are environmentally friendly and also have potential high yields for particular types of nanoparticles.

## Figures and Tables

**Table 1:** Particular techniques for preparation of selected noble metals nanoparticles and their typical size distributions.

Type of NPs					Synthetic Method	NP Size [nm]	References
Au	Ag	Pd	Pt	Cu			
x					Sol–gel micro reactors	5–50	[61,62]
x	x	x	x		PVD into liquid substrate	2–10	[33,63,64]
		x	x		Reduction in acidic environment	3–40	[65]
x					Reduction process	2–40	[66,67,68,69,70]
x				x	γ-Irradiation	3–30	[71]
				x	pH control of Cu complexes	48–150	[72]
x	x	x			Biosynthesis	9–25	[73,74,75,76,77]
	x				Wet chemistry	20–60	[78,79]
